# Metabolomic dysregulation in the association between major depressive disorder and chronic pancreatitis: a large prospective cohort study

**DOI:** 10.3389/fnut.2026.1891809

**Published:** 2026-09-10

**Authors:** Weizhen Yang, Changgan Chen, JunPeng Li, Guochen Li, Dong Ning, Ziyue Wang, Sanbing Shen, Yaqiong Liu, Lin Cheng

**Affiliations:** 1 Department of General Medicine, The Second Xiangya Hospital of Central South University, Changsha, China; 2 Department of General Surgery, Fujian Medical University Union Hospital, Fuzhou, China; 3 Department of Surgery, Tohoku University Graduate School of Medicine, Sendai, Japan; 4 Department of Epidemiology, NHC Key Laboratory for Health Technology Assessment, Fudan University School of Public Health, Shanghai, China; 5 Regenerative Medicine Institute (REMEDI), Biomedical Sciences Building, School of Medicine, University of Galway, Galway, Ireland; 6 School of Nursing and Midwifery, University of Galway, Galway, Ireland; 7 Postdoctoral Mobile Station of Clinical Medicine, The Second Xiangya Hospital of Central South University, Changsha, China

**Keywords:** chronic pancreatitis, cohort study, major depressive disorder, mediation analysis, metabolomics

## Abstract

**Background:**

Major depressive disorder (MDD) is increasingly recognized as a systemic condition accompanied by metabolic disturbances, particularly in lipid metabolism. However, whether MDD is associated with the development of chronic pancreatitis (CP), a chronic inflammatory disease with substantial metabolic and nutritional consequences, remains unclear. We aimed to investigate the prospective association between MDD and incident CP and to assess the potential mediating role of circulating nuclear magnetic resonance (NMR)-based metabolic biomarkers.

**Methods:**

In this prospective study of 273,524 UK Biobank participants, MDD was identified using hospital records and self-reports. Incident CP was ascertained through linked health records. Cox proportional hazards models were used to estimate associations, and mediation analyses based on NMR metabolomics were performed. A metabolomic signature was further derived using least absolute shrinkage and selection operator (LASSO) regression.

**Results:**

Over a median follow-up of 13.7 years, 411 incident CP cases were identified. MDD was independently associated with an increased risk of CP (HR 1.47, 95% CI 1.10–1.96). Exploratory mediation analyses suggested that lipid-related metabolites accounted for part of the observed association, including triglycerides in HDL particles (HDL-TG; 5.10%), triglycerides in LDL particles (LDL-TG; 4.19%), and fatty acid unsaturation (7.61%). A 51-metabolite signature explained 20.80% (95% CI 13.20–27.20%) of the association. The association was stronger in participants aged ≤60 years (*P*-interaction = 0.014).

**Conclusion:**

Major depressive disorder was associated with a higher risk of CP, and exploratory mediation analyses suggested that lipid related metabolomic alterations may account for part of this association. These findings highlight metabolic dysregulation as a potential biological correlate of the observed MDD–CP association.

## 1 Introduction

Chronic pancreatitis is a progressive and irreversible inflammatory disease of the pancreas characterized by persistent abdominal pain, exocrine and endocrine pancreatic insufficiency, and multiple metabolic disturbances (1, 2). As the disease progresses, patients frequently develop malnutrition, impaired glucose metabolism, and substantial reductions in quality of life, imposing a considerable long-term burden on healthcare systems (3, 4). Epidemiological studies have shown that the prevalence of CP has been steadily increasing in many countries (4–6). Although alcohol consumption, smoking, and genetic susceptibility are recognized as the major risk factors, these factors alone cannot fully explain the observed disease burden (1, 2, 7). Therefore, identifying novel risk factors and elucidating their underlying biological mechanisms are essential for improving early prevention and risk management of CP.

Major depressive disorder (MDD) is one of the most common psychiatric disorders worldwide, affecting approximately 3% of the adult population (8). Increasing evidence suggests that depression is not only a mental health condition but is also closely associated with a range of chronic systemic diseases, including cardiovascular disease (9), diabetes (10), and metabolic syndrome (11). Although previous studies have explored the impact of depression on certain gastrointestinal disorders such as gastroesophageal reflux disease (12), its potential role in the development of CP has received little attention (13). From a biological perspective, depression can disrupt physiological homeostasis through multiple pathways, including chronic low grade inflammation, activation of the neuroendocrine stress axis, and dysregulation of metabolic processes (14–16). Notably, these mechanisms are also critically involved in the pathogenesis and progression of CP (17, 18). However, large prospective population based studies systematically evaluating the longitudinal association between MDD and CP remain lacking. Although recent Mendelian randomization (MR) studies have provided genetic evidence for a potential relationship between depression and CP (13, 19), they evaluate genetically proxied liability rather than clinically observed MDD and do not directly characterize incident disease risk or measured circulating metabolic pathways in a longitudinal population-based setting. Prospective cohort studies integrating detailed metabolic profiling are therefore needed to complement the existing genetic evidence.

Advances in metabolomics have provided new tools for investigating the systemic biological links between psychiatric disorders and somatic diseases. A large pooled analysis using nuclear magnetic resonance metabolomics measured 230 metabolic measures in 5,283 individuals with depression and 10,145 controls. Depression was associated with a distinct circulating lipid profile characterized by higher VLDL- and triglyceride-related measures, including triglycerides within HDL and LDL particles, and lower levels of several HDL related measures (20, 21). These findings suggest that depression related metabolic abnormalities extend beyond individual metabolites and may reflect broader remodeling of lipid transport and fatty acid metabolism. Mechanistically, this lipid profile may be related to depression associated neuroendocrine and inflammatory dysregulation. Hypothalamic–pituitary–adrenal (HPA) axis dysregulation and chronic low grade inflammation are common biological features of stress related depressive disorders (14–16). Altered glucocorticoid signaling and inflammation related insulin resistance may promote adipose tissue lipolysis and free fatty acid release and, by increasing hepatic fatty acid flux, favor VLDL production (22, 23). Inflammation may further alter HDL composition and function (24). Similar lipid disturbances may also be relevant to pancreatic injury. Excess triglyceride rich lipoproteins and their hydrolysis products may promote the accumulation of unbound free fatty acids, mitochondrial dysfunction, and pro-inflammatory signaling, thereby contributing to pancreatic acinar cell injury (25, 26). The degree of fatty acid unsaturation may also influence the intensity of inflammatory responses, and polyunsaturated fatty acids have been shown to reduce inflammation and tissue injury in experimental pancreatitis (27, 28). Metabolomics may therefore help identify potential intermediate biological phenotypes linking MDD with pancreatic inflammation. However, whether these metabolic alterations mediate the prospective association between MDD and CP remains unclear (19, 29).

Against this background, the present study leveraged data from 273,524 participants in the UK Biobank prospective cohort to investigate the longitudinal association between major depressive disorder and the risk of incident CP. Furthermore, we integrated nuclear magnetic resonance (NMR) based metabolomics data to identify metabolic biomarkers associated with depression and to quantify their potential mediating roles in this association through mediation analysis. In addition, we constructed an integrated metabolic signature using LASSO regression to evaluate the contribution of overall metabolic patterns to the relationship between MDD and CP. By combining prospective epidemiological and metabolomic approaches, this study aims to complement existing genetic evidence and provide new insights into the population level association and potential metabolic pathways linking MDD with CP.

## 2 Materials and methods

### 2.1 Study design and participants

The UK Biobank is a large-scale prospective cohort comprising over 500,000 participants aged 37–73 years, recruited across England, Scotland, and Wales between 2006 and 2010. At enrollment, subjects underwent comprehensive baseline evaluations consisting of touchscreen questionnaires, brief interviews, physical measurements, and biospecimen collection (30). By integrating with national health records, such as primary care data, hospital admissions, and mortality registries—the project enables long-term tracking of health outcomes for NHS-registered participants who provided informed consent for follow-up (31).

The current study included participants who were free of pancreas disease at baseline. After the exclusion of those who had missing data on metabolomics measurement (*n* = 227,309), or those who withdrew informed consent subsequently (*n* = 158), a total of 273,524 participants were eligible for final analyses.

### 2.2 Assessment of exposure and outcome

In this study, MDD was ascertained using linked hospital inpatient records based on the International Classification of Diseases, 10th Revision (ICD-10) codes F32 (depressive episode) and F33 (recurrent depressive disorder) (32, 33), together with self-reported physician-diagnosed depression. The UK Biobank-derived probable major depression phenotype based on questionnaire data was not used in the present study. For the primary analysis, F32 and F33 were combined as a single MDD exposure. In an ancillary analysis restricted to participants with ICD-coded depression, F32 and F33 were also examined separately to assess associations according to depressive episode type. Detailed and consistently available information on depression severity, episode duration, and episode frequency was not available across the full analytic cohort and was therefore not incorporated into the present analysis.

In accordance with established protocols from the UK Biobank and prior research (34, 35), cases of CP were identified through linked hospital admission records and mortality data using ICD-10 codes K86.0 and K86.1. Participants were followed from the date of their initial assessment until the earliest occurrence of CP, death, or the end of follow-up (31 October 2022 for England, 31 May 2022 for Wales, and 31 August 2022 for Scotland).

### 2.3 NMR metabolomics

The EDTA plasma samples were collected during the UK Biobank baseline assessment between 2006 and 2010 and stored at −80 °C until NMR analysis. Only metabolomic measurements derived from baseline plasma samples were included. The measurements took place between June 2019 and April 2020 (Phase 1), April 2020 and June 2022 (Phase 2) and September 2022 and November 2023 (Phase 3) using 9 spectrometers at Nightingale Health, based in Finland. The metabolomics data used in this study were accessed in October 2024.

Metabolic profiling was performed using Nightingale Health’s high throughput proton nuclear magnetic resonance (^1H-NMR) spectroscopy platform, for which the analytical methods and quality control procedures have been described previously (36). Frozen EDTA plasma samples were thawed overnight at 4 °C, gently mixed, and centrifuged at 3,400 × g for 3 min at 4 °C. Plasma was then mixed with phosphate buffer at a 1:1 ratio and transferred into 3-mm NMR tubes. Two spectra were acquired for each sample using a 500-MHz Bruker AVANCE III HD spectrometer: a presaturated proton NMR spectrum, primarily capturing protein and lipoprotein lipid signals, and a T2-relaxation-filtered spectrum, enhancing the detection of low-molecular-weight metabolites. Metabolic measures were quantified using Nightingale Health’s automated proprietary software.

The platform provided 249 standard metabolic measures, including 168 absolute level measures and 81 derived ratios. The present study included all 168 absolute level measures, covering lipoprotein lipid composition, fatty acid profiles, amino acids, ketone bodies, and glycolysis related metabolites. For each of 14 lipoprotein subclasses, triglycerides, phospholipids, total cholesterol, cholesteryl esters, free cholesterol, and total lipids were quantified separately. Most concentration-based measures were reported in mmol/L, whereas mean lipoprotein particle diameters and the degree of fatty acid unsaturation were reported in nm and degrees, respectively.

“Triglycerides in HDL particles” (HDL-TG; UK Biobank Field 23,410) and “triglycerides in LDL particles” (LDL-TG; Field 23,409) represent the triglyceride concentrations carried within the corresponding HDL and LDL fractions and are distinct from HDL cholesterol (HDL-C; Field 23,406) and LDL cholesterol (LDL-C; Field 23,405).

Analytical quality control included automated spectral assessment, real time monitoring of measurement consistency within and across spectrometers, two internal control samples per 96 well plate, and two blinded UK Biobank duplicate samples per plate. Prespecified coefficient-of-variation criteria were met across consecutive batches, with coefficients of variation below 5% for many metabolic measures (37). Additional preprocessing, including log transformation, standardization, and imputation of missing metabolite values, is described in Section 2.5. The full list of included metabolic measures, together with their corresponding UK Biobank field IDs is provided in Supplementary Table 1.

### 2.4 Covariates

Covariates were selected based on prior knowledge and previous studies (34, 38). The covariates included sociodemographic characteristics, lifestyle factors, anthropometric measures, and comorbidities.

Sociodemographic variables included age (continuous), sex (men or women), ethnicity (White or other ethnic groups), education level (college or university degree, NVQ/HNC or equivalent, other professional qualification, A-level/AS-level or equivalent, and O-level/GCSE or equivalent), annual household income (<£18,000, £18,000–£30,999, £31,000–£51,999, £52,000–£100,000, >£100,000), and the Townsend deprivation index.

Lifestyle factors were self-reported and included smoking status (never, former, or current), alcohol intake (never, former, or current), and physical activity. Total physical activity was represented by the UK Biobank-derived summed metabolic equivalent task (MET) minutes per week measure (Field 22,040), which combines walking, moderate-intensity activity, and vigorous-intensity activity. According to the International Physical Activity Questionnaire (IPAQ) scoring framework used for derivation of these MET measures, walking, moderate-intensity activity, and vigorous-intensity activity are assigned values of 3.3, 4.0, and 8.0 METs, respectively, and activity-specific MET-minutes/week are calculated as the MET value × minutes per day × days per week and summed across activity types. For the present analysis, total physical activity was categorized as inactive (<600 MET-min/week), moderately active (600–<3,000 MET-min/week), or highly active (≥3,000 MET-min/week). Further details on the derivation of the MET measures and IPAQ scoring are available through the UK Biobank Showcase (Field 22,040) (30).

Anthropometric measurements were obtained at the baseline assessment center. Body mass index (BMI) was calculated using body weight and standing height measured at the baseline assessment center visit, rather than self-reported usual or historical weight. BMI was calculated as measured body weight in kilograms divided by standing height in meters squared (kg/m^2^) and categorized according to World Health Organization criteria (39) as underweight (<18.5), normal weight (18.5–<25), overweight (25–<30), and obese (≥30).

Baseline diabetes mellitus was the principal comorbidity covariate included in the primary Cox model. Information on baseline use of hypoglycemic and lipid-lowering medications was also available and was summarized descriptively, but these medication variables were not included in the prespecified primary adjustment model. Several additional pancreatitis-related clinical and lifestyle factors were considered as potential confounders but were not included in the primary model because they could not be consistently or comprehensively ascertained across the full analytical cohort. These included gallstone disease, autoimmune disorders, prior pancreatic surgery, chronic opioid exposure, family history of pancreatitis, clinical hyperlipidemia, and detailed dietary habits. Gallstone disease, autoimmune disorders, and prior pancreatic surgery could be identified from linked hospital records; however, their ascertainment depended on recorded diagnoses or procedures and was therefore incomplete. Chronic opioid exposure could not be reliably characterized because sufficiently detailed longitudinal medication data were not consistently available across the full cohort, and a specific and consistently available measure of family history of pancreatitis was unavailable. Clinical hyperlipidemia was not included as a binary diagnosis because detailed lipid related phenotypes were evaluated separately in the metabolomic analyses. Detailed dietary variables were also not included because dietary information was not consistently available across the full analytical cohort. Residual confounding from these incompletely measured or unavailable clinical and lifestyle factors therefore cannot be excluded.

### 2.5 Statistical analysis

Baseline characteristics were summarized as percentages for categorical variables and medians with interquartile ranges (IQRs) for continuous variables. Differences between the MDD and non-MDD groups were compared using Wilcoxon rank-sum tests for continuous variables and chi-square tests for categorical variables.

Cox proportional hazards models were used to estimate hazard ratios (HRs) and 95% confidence intervals (CIs) for the association between MDD and incident CP. The proportional hazards assumption was assessed using scaled Schoenfeld residuals. Two models were constructed: a minimally adjusted model (Model 1) including age, sex, and ethnicity, and a fully adjusted model (Model 2) additionally adjusted for Townsend deprivation index, household income, education level, smoking status, alcohol intake, BMI, physical activity, and baseline diabetes.

The proportions of missing data for the covariates were as follows: age, 0%; sex, 0%; ethnicity, 0.45%; Townsend deprivation index, 0.12%; household annual income, 4.61%; education level, 1.16%; smoking status, 0.49%; alcohol intake, 0.23%; physical activity, 22.8%; body mass index, 0.37%; and baseline diabetes, 0%. The corresponding numbers and percentages of missing observations are provided in Supplementary Table 2. Missing values in continuous covariates were handled using multiple imputation, whereas categorical covariates with missing values were assigned to a separate missing category.

Metabolite concentrations were log-transformed and standardized before analysis. Missing metabolite values were imputed using the mean of the corresponding metabolite, and metabolite-specific missingness is reported in Supplementary Table 1.

To identify candidate mediating metabolites, multiple linear regression analyses were first performed to determine metabolites significantly associated with MDD (adjusted as in Model 2). Subsequently, Cox proportional hazards models were applied to these MDD-associated metabolites to identify those also associated with incident CP (adjusted as in Model 2). Metabolites meeting both criteria were included in subsequent mediation analyses.

**Figure 1:**
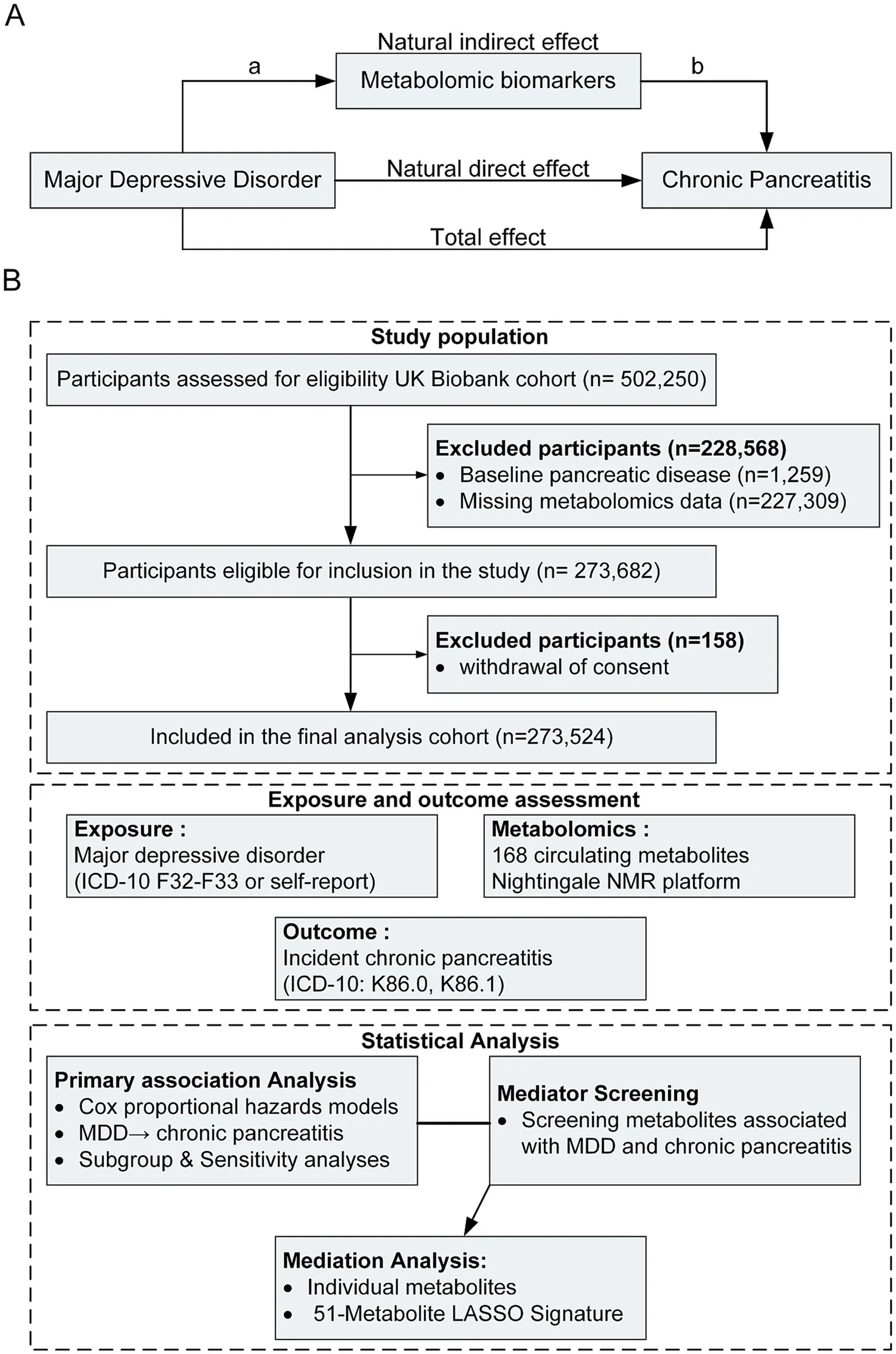
Study framework and participant selection. **(A)** Conceptual framework of the mediation analysis. **(B)** Flowchart of the study population and analytical pipeline.

Mediation analyses were conducted using the R package mediation to quantify the proportion of metabolic mediation in the association between MDD and incident CP. Confidence intervals were estimated using the quasi-Bayesian Monte Carlo method with 1,000 simulations (40). The total effect was decomposed into the natural direct effect (NDE; MDD → CP) and the natural indirect effect (NIE; MDD → metabolites → CP), as illustrated in Figure 1A.

To derive an integrated metabolomic signature associated with MDD, LASSO regression was applied using the 168 circulating metabolites as candidate predictors with MDD status as the outcome. The penalty parameter was selected using 10-fold cross-validation within the full analytical cohort. The metabolomic signature was calculated as the weighted sum of the metabolites retained by the LASSO model, with weights corresponding to the estimated regression coefficients. No separate training/test split or independent holdout sample was used for out-of-sample evaluation; therefore, cross-validation was used for model tuning rather than as an independent validation of the final signature. Because metabolite selection, signature construction, and subsequent mediation analysis were conducted within the same analytical cohort, the apparent mediated proportion could be subject to in-sample optimism. To assess and reduce this potential optimism, 1,000 bootstrap resamples were used to obtain a bootstrap-adjusted estimate of the mediated proportion. The metabolomic signature was then evaluated using the same mediation framework described above.

Stratified analyses were conducted according to clinically relevant subgroups, including age (≤60 or >60 years), sex (male or female), alcohol intake (never, past, or current), and diabetes status (yes or no). Interactions between MDD and subgroup variables were evaluated by including interaction terms in multivariable Cox models, and statistical significance was assessed using likelihood ratio tests.

Several sensitivity analyses were conducted to evaluate the robustness of the findings. First, to minimize potential reverse causation, analyses were repeated after excluding incident CP cases occurring within the first 2 years of follow-up. An additional 5-year lag analysis was also conducted to further assess the potential influence of subclinical pancreatic disease present at baseline. Second, Fine–Gray competing risk models were used to estimate subdistribution hazard ratios while treating all-cause and other-cause deaths as competing events. Third, to address potential bias due to missing covariate data, multiple imputation using random forest–based chained equations was performed and analyses were repeated (41). Fourth, users of antidepressant medications were considered potential MDD cases and added to the exposed group. Finally, additional analyses were performed after excluding participants using lipid-lowering medications.

All statistical analyses were conducted using SAS version 9.4 (SAS Institute, Cary, NC, United States) and R software (version 4.3.1). A two-sided *p*-value <0.05 was considered statistically significant. For analyses involving circulating metabolites, statistical significance was defined as a Benjamini–Hochberg false discovery rate (FDR) < 0.05.

### 2.6 Ethics approval

The UK Biobank received ethical approval from the North West Multi centre Research Ethics Committee (Reference No: 11/NW/0382), which covers all research using the UK Biobank resource. This study was conducted under UK Biobank Application Number 142341. All participants provided written informed consent at recruitment, and the study was performed in accordance with the Declaration of Helsinki.

## 3 Results

### 3.1 Participants selection

The study cohort was derived from the UK Biobank, initially comprising 502,250 participants. Figure 1B depicts the flowchart for participant selection and the systematic analytical pipeline employed in this study. To ensure focus on incident CP, 1,259 individuals with prevalent pancreatitis or pancreatic disease at baseline were excluded. Additionally, 227,309 participants lacking complete metabolomics data were removed. This yielded 273,682 eligible participants. After further excluding 158 individuals who withdrew consent, the final analytical sample included 273,524 participants.

### 3.2 Baseline characteristics

Table 1 summarizes the baseline characteristics of the 273,524 participants according to MDD status. Among them, 24,401 (8.92%) participants were classified as having MDD, while 249,123 (91.08%) were in the non-MDD group. Compared with participants without MDD, those with MDD were generally younger (median age 56 vs. 58 years) and included a lower proportion of males. The proportion of White individuals was slightly higher in the MDD group. With respect to socioeconomic characteristics, participants with MDD tended to have lower socioeconomic status. They had higher Townsend deprivation index values and a greater proportion of individuals in the lowest household income category (<£18,000). Additionally, a slightly higher proportion of individuals without formal qualifications was observed in the MDD group. Regarding lifestyle factors, individuals with MDD showed less favorable behavioral profiles. They had a higher prevalence of current smoking and a lower proportion of current alcohol consumption compared with those without MDD. Participants with MDD were also less physically active, with a greater proportion falling into the lowest physical activity category (<600 MET-min/week). In terms of clinical and metabolic characteristics, the MDD group exhibited a higher prevalence of overweight or obesity, diabetes, and use of hypoglycemic and lipid-lowering medications. All baseline differences between the two groups were statistically significant (*p* < 0.0001). Overall, participants with MDD had a greater burden of measured metabolic and behavioral abnormalities at baseline.

**Table 1:** Baseline characteristics of the study participants according to the MDD status.

Characteristics	Overall	Non-MDD	MDD	*p*-value
Count	273,524	249,123	24,401	
Age (median [IQR])	58.000 [50.000, 63.000]	58.000 [50.000, 63.000]	56.000 [49.000, 62.000]	<0.0001
Sex of male (%)	125,875 (46.02)	117,464 (47.15)	8,411 (34.47)	<0.0001
Ethnic (%)				
White	258,876 (94.64)	235,435 (94.51)	23,441 (96.07)	<0.0001
Others	13,425 (4.91)	12,573 (5.05)	852 (3.49)	
Townsend deprivation index (median [IQR])	−2.200 [−3.680, 0.440]	−2.240 [−3.700, 0.340]	−1.690 [−3.420, 1.430]	<0.0001
Annual household income (£) (%)				
<18,000	54,341 (19.87)	47,104 (18.91)	7,237 (29.66)	<0.0001
18,000–30,999	60,297 (22.04)	54,793 (21.99)	5,504 (22.56)	
31,000-51,999	60,504 (22.12)	55,769 (22.39)	4,735 (19.40)	
52,000-100,000	46,316 (16.93)	43,478 (17.45)	2,838 (11.63)	
>100,000	11,816 (4.32)	11,250 (4.52)	566 (2.32)	
Education level (%)				
College or university degree	86,578 (31.65)	79,323 (31.84)	7,255 (29.73)	<0.0001
NVQ or HNC or equivalent	29,965 (10.96)	27,226 (10.93)	2,739 (11.22)	
Other professional qualification	73,277 (26.79)	66,717 (26.78)	6,560 (26.88)	
Advanced /Advanced subsidiary or equivalent	18,381 (6.72)	16,812 (6.75)	1,569 (6.43)	
CSE or Ordinary level/GCSE or equivalent	14,227 (5.20)	12,991 (5.21)	1,236 (5.07)	
No qualification	47,916 (17.52)	43,150 (17.32)	4,766 (19.53)	
Alcohol intake (%)				
Never	11,869 (4.34)	10,711 (4.30)	1,158 (4.75)	<0.0001
Past	9,660 (3.53)	7,918 (3.18)	1742 (7.14)	
Current	251,356 (91.90)	229,935 (92.30)	21,421 (87.79)	
Smoking status (%)				
Never	148,792 (54.40)	137,005 (54.99)	11,787 (48.31)	<0.0001
Past	94,665 (34.61)	86,094 (34.56)	8,571 (35.13)	
Current	28,736 (10.51)	24,806 (9.96)	3,930 (16.11)	
BMI (%)				
Normal weight	88,082 (32.20)	81,142 (32.57)	6,940 (28.44)	<0.0001
Underweight	1,352 (0.49)	1,229 (0.49)	123 (0.50)	
Overweight or obese	183,067 (66.93)	165,843 (66.57)	17,224 (70.59)	
MET (%)				
<600 min	38,989 (14.25)	34,651 (13.91)	4,338 (17.78)	<0.0001
600–3,000 min	106,206 (38.83)	97,448 (39.12)	8,758 (35.89)	
>3,000 min	65,972 (24.12)	60,803 (24.41)	5,169 (21.18)	
Use of hypoglycemic agents (%)	10,088 (3.69)	8,975 (3.60)	1,113 (4.56)	<0.0001
Use of lipid-lowering medication (%)	50,192 (18.35)	45,100 (18.10)	5,092 (20.87)	<0.0001
Diabetes (%)	15,286 (5.59)	13,555 (5.44)	1731(7.09)	<0.0001

Data presented as frequency (%) or median (Q25–Q75). *P* for ANOVA or Wilcoxon tests (continuous variables) or Chi-squared tests (categorical variables).

NVQ, National Vocational Qualification; HNC, Higher National Certificate; CSE, Certificate of Secondary Education; GCSE, General Certificate of Secondary Education.

### 3.3 Association between MDD and CP

**Figure 2:**
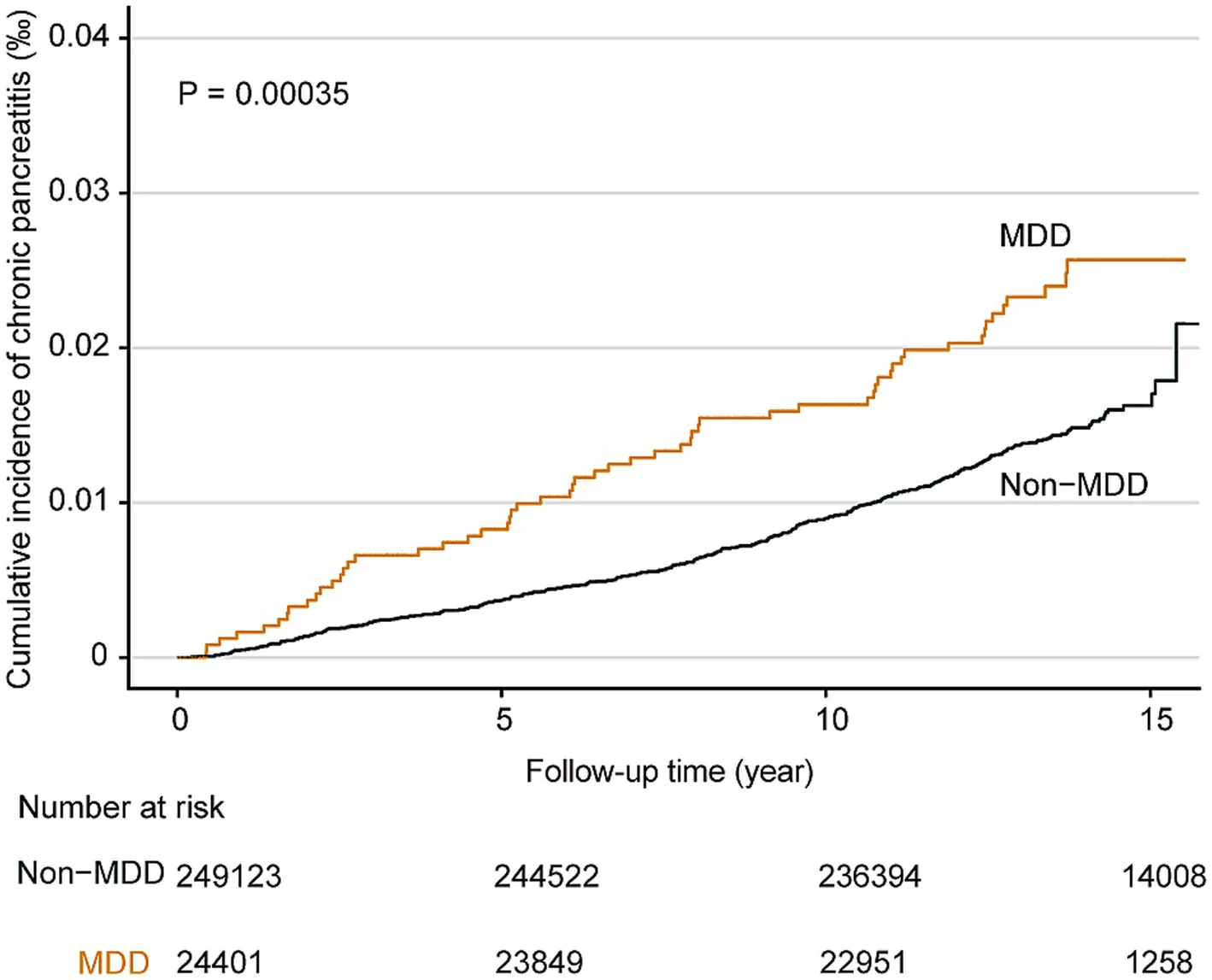
Cumulative incidence of chronic pancreatitis among individuals with and without MDD during a 15-year follow-up period.

Figure 2 shows the cumulative incidence of CP according to MDD status during the 15-year follow-up period. Participants with MDD consistently exhibited a higher cumulative incidence of CP compared with those without MDD.

We estimated multivariable adjusted hazard ratios together with crude incidence rates to provide both relative and absolute event-rate context. The crude incidence rates of CP were approximately 18 and 11 cases per 100,000 person-years among participants with and without MDD, respectively, corresponding to a crude incidence rate difference of approximately 7 additional cases per 100,000 person-years. Thus, although MDD was associated with a higher relative risk of CP, the absolute excess incidence was small. In the Cox proportional hazards model adjusted for age, sex, and ethnicity (Model 1), MDD was significantly associated with a higher risk of incident CP (HR 1.954, 95% CI 1.475–2.589). After further adjustment for socioeconomic factors, lifestyle behaviors, BMI, physical activity, and baseline diabetes (Model 2), the association was attenuated but remained statistically significant (HR, 1.468; 95% CI, 1.101–1.955) (Table 2). No evidence of violation of the proportional hazards assumption was observed in any of the Cox regression models based on scaled Schoenfeld residuals. The attenuation from Model 1 to Model 2 suggests that shared metabolic and behavioral characteristics may partly contribute to the observed association, although a significant association persisted after adjustment for these measured factors.

**Table 2:** Associations between major depressive disorder and the risk of chronic pancreatitis.

MDD status	Incidence (per 100,000 person-years)	Cases	Chronic Pancreatitis	
			HR1 (95% CI)	HR2 (95% CI)
Major depressive disorder	12	411		
No	11	354	1.000 (reference)	1.000 (reference)
Yes	18	57	1.954 (1.475–2.589)	1.468 (1.101–1.955)

HR1 (95%CI): adjusted for age, sex, and ethnicity.

HR2 (95%CI): adjusted for age, sex, ethnicity, Townsend deprivation index, household income, highest education, smoking status, alcohol intake, body mass index, physical activity and baseline diabetes.

Major depressive disorder determined based on hospital inpatient admission records and self-report.

In an ancillary analysis restricted to participants with ICD-coded depression, the fully adjusted association with incident CP was observed for both depressive episode (F32; HR 1.442, 95% CI 1.078–1.929) and recurrent depressive disorder (F33; HR 1.699, 95% CI 1.240–2.327).

We further conducted subgroup analyses stratified by age, sex, alcohol intake, and diabetes status (Supplementary Table 3). A significant interaction was observed for age (*P* for interaction = 0.014). Specifically, the association between MDD and CP was stronger among participants aged ≤60 years (HR2 1.786, 95% CI 1.235–2.583), whereas no significant association was observed among those aged >60 years (HR2 1.103, 95% CI 0.685–1.775). In sex-stratified analyses, MDD was associated with an increased risk of CP in males (HR2 1.507, 95% CI 1.038–2.188), whereas the association in females did not reach statistical significance (HR2 1.393, 95% CI 0.888–2.186). When stratified by alcohol intake, a significant association was observed among current drinkers (HR2 1.441, 95% CI 1.068–1.944), but not among never or past drinkers (HR2 1.904, 95% CI 0.623–5.824). Similarly, the association was significant among participants without diabetes (HR2 1.499, 95% CI 1.089–2.062) but not among those with diabetes (HR2 1.350, 95% CI 0.699–2.610).

To evaluate the robustness of the findings, several sensitivity analyses were conducted (Supplementary Table 4). After excluding CP cases occurring within the first 2 years of follow-up, the association remained statistically significant (HR2 1.420, 95% CI 1.050–1.921). The association was also materially consistent in an additional 5-year lag analysis (HR 1.429, 95% CI 1.049–1.947). Consistent results were observed when applying the Fine–Gray competing risk model (HR2 1.452, 95% CI 1.092–1.929) and when handling missing covariate data using multiple imputation (HR2 1.478, 95% CI 1.110–1.968). In addition, when users of antidepressant medications were considered potential MDD cases and included in the exposed group, the association remained robust (HR2 1.558, 95% CI 1.209–2.008).

### 3.4 Mediation effects of metabolic biomarkers and metabolic signature

**Figure 3:**
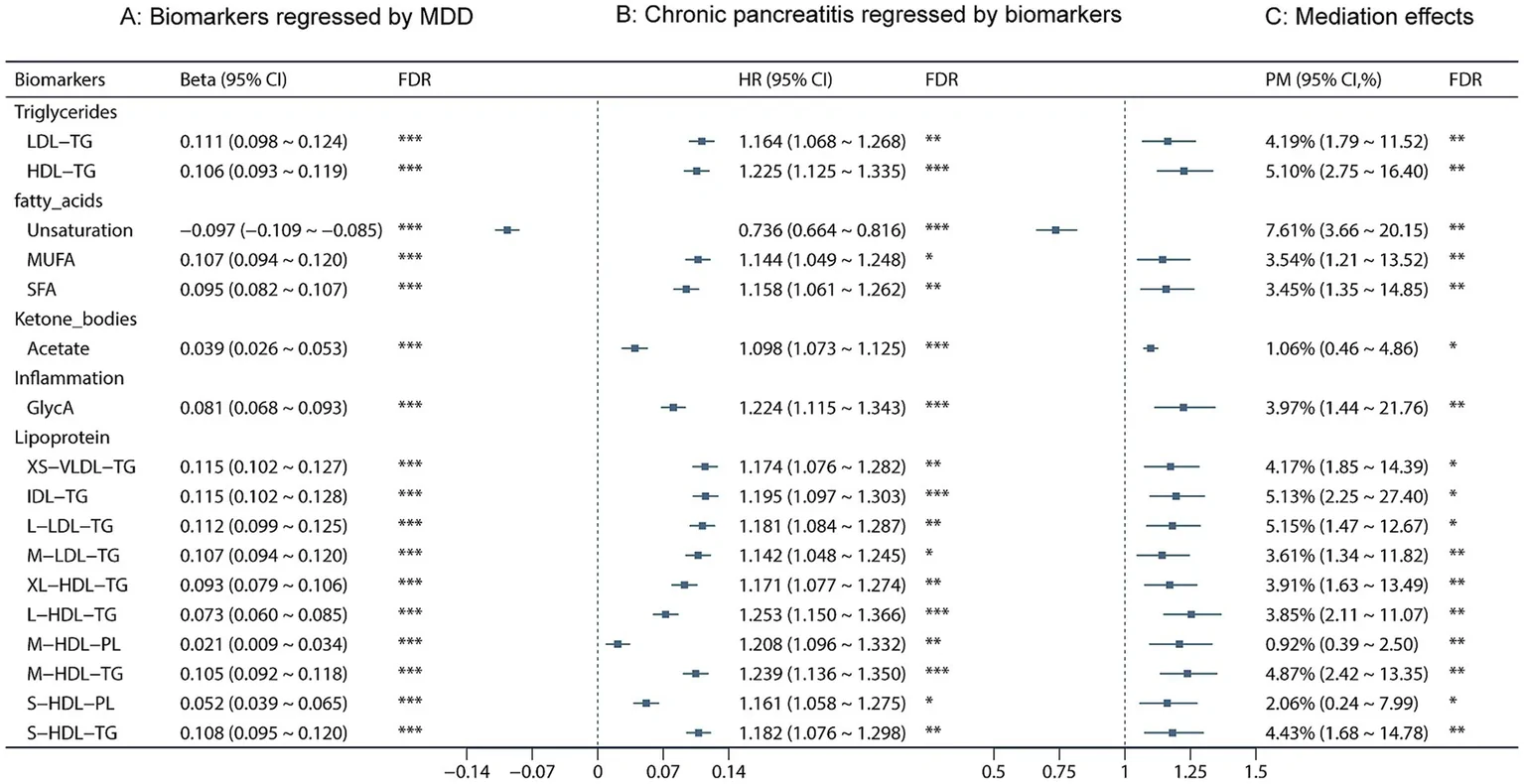
Mediation analysis of circulating metabolites in the association between MDD and incident CP. **(A)** Associations of MDD on selected metabolic biomarkers. **(B)** Associations between biomarkers and risk of CP. **(C)** Proportions of the association mediated by individual metabolites. CI, confidence intervals; HR, hazard ratios; FDR, false discovery rate; PM, proportion mediated. Statistical significance was defined as a Benjamini–Hochberg false discovery rate (FDR): *p < 0.05, **p < 0.01, ***p < 0.001.

MDD was associated with altered lipids and inflammatory profiles, including higher HDL-TG and LDL-TG concentrations, a lower degree of fatty acid unsaturation, and elevated levels of glycoprotein acetyls and acetate (Supplementary Table 5). These biomarkers were, in turn, linked to the risk of CP (Supplementary Table 6). Figure 3 illustrates these relationships across three panels.

Panel A depicts the associations of MDD with selected biomarkers, showing significant increases in LDL-TG (beta = 0.111,95% CI: 0.098–0.124), HDL-TG (beta = 0.106, 95% CI: 0.093–0.119), glycoprotein acetyls (beta = 0.081, 95% CI: 0.068–0.093), and acetate (beta = 0.039, 95% CI: 0.026–0.053), along with a decrease in the degree of unsaturated fatty acids (beta = −0.097, 95% CI: −0.109 to −0.085).

Panel B presents the associations between these biomarkers and CP risk: HDL-TG (HR = 1.225, 95% CI: 1.125–1.335, *FDR < 0.001*) and LDL-TG (HR = 1.164, 95% CI: 1.068–1.268, *FDR < 0.001*) were associated with higher risk, whereas a higher degree of fatty acid unsaturation was associated with a lower risk of CP (HR = 0.736, 95% CI: 0.664–0.816, *FDR < 0.001*).

Panel C quantifies the mediation effects, showing that HDL-TG mediated 5.10% (95% CI: 2.75–16.40, *FDR < 0.01*), LDL-TG mediated 4.19% (95% CI: 1.79–11.52, *FDR < 0.05*), and unsaturated fatty acids mediated 7.61% (95% CI: 3.66–20.15, *FDR < 0.01*) of the MDD-CP association, with smaller contributions from saturated fatty acids (3.45%), glycoprotein acetyls (3.97%), and acetate (1.06%).

Following the sensitivity analyses that excluded individuals with newly diagnosed cases within 2 years or those using lipid-lowering or hypoglycemic medications, the associations between MDD, the identified biomarkers, and incident CP remained statistically significant (Supplementary Table 7).

A total of 51 metabolites were selected to develop metabolic signature for MDD through LASSO regression. The selected metabolites and their corresponding regression coefficients are summarized in Supplementary Table 8, with approximately 45% lipoprotein-related measures, 4% lipids, 12% fatty acids, and 40% amino acids and other metabolites. MDD was significantly associated with an increase in metabolic signature score (beta = 0.065, *p* < 0.001). Additionally, a higher metabolic signature score was independently associated with an elevated risk of incident CP (HR = 2.776, 95% CI: 2.275–3.388). Exploratory mediation analysis estimated that the metabolomic signature accounted for 20.80% (95% CI: 13.20–27.20) of the observed association between MDD and CP, with the bootstrap-adjusted estimate decreasing to 19.2%.

## 4 Discussion

This study included 273,524 participants from the UK Biobank who were followed for a median of 13.7 years and identified a prospective association between MDD and incident CP. After adjustment for age, sex, and ethnicity, participants with MDD had an approximately 95% higher hazard of CP than those without MDD (HR, 1.954; 95% CI, 1.475–2.589). Participants with MDD also exhibited a less favorable metabolic and behavioral profile at baseline, including higher prevalences of overweight or obesity, diabetes, and current smoking. After further adjustment for socioeconomic, lifestyle, and metabolic factors, the association was attenuated but remained statistically significant (HR, 1.468; 95% CI, 1.101–1.955), suggesting that shared metabolic and behavioral characteristics may explain part, but not all, of the observed association. The association also remained consistent across multiple sensitivity and competing risk analyses. In absolute terms, the crude incidence rates of CP were approximately 18 and 11 cases per 100,000 person-years among participants with and without MDD, respectively, corresponding to approximately 7 additional cases per 100,000 person-years. Thus, although the relative association was statistically significant, the absolute excess incidence at the individual level was small. Nevertheless, given the high prevalence of MDD, the substantial and often irreversible long term burden of CP, including chronic pain and pancreatic insufficiency, and the limited prospective evidence linking psychiatric disorders with pancreatic disease, this association may still have clinical and public health relevance at the population level. From a clinical perspective, however, the modest relative association and low absolute incidence do not currently support routine screening for CP solely on the basis of MDD status. Likewise, the observational and exploratory nature of the metabolomic findings does not establish that lipid modifying interventions would reduce CP risk in individuals with MDD. Rather, our findings suggest that the clinical management of individuals with MDD may benefit from a more integrated assessment of established pancreatic related and metabolic risk factors, particularly because modifiable factors such as smoking, alcohol consumption, obesity, diabetes, and metabolic abnormalities were more common in this population and may contribute to the observed association with CP. In addition, stratified analyses showed a stronger association among participants aged ≤60 years (HR, 1.79; 95% CI, 1.24–2.58), although this subgroup finding should be considered hypothesis generating and requires confirmation in independent populations before informing age specific clinical practice.

Evidence on the relationship between psychiatric disorders and pancreatic diseases remains limited. Previous studies have mainly comprised a small number of Mendelian randomization analyses, whereas large prospective studies and systematic evaluations of shared biological pathways are still lacking (13, 19). Previous MR studies have provided valuable genetic evidence regarding the potential relationship between MDD and CP. However, because MR evaluates associations based on genetically proxied liability, it does not directly characterize the longitudinal association between observed MDD and incident CP in a population-based setting, nor does it quantify absolute disease burden or measured circulating metabolic pathways that may accompany this association. In contrast, our 13.7 year prospective design provides longitudinal temporal ordering, with MDD status assessed before incident CP, and allows estimation of incidence, subgroup specific associations, and robustness across sensitivity and competing-risk analyses. Furthermore, by integrating NMR based metabolomics, we were able to explore whether circulating metabolic alterations may account for part of the observed association. Specifically, we quantified the contributions of individual lipid related measures, including HDL-TG, LDL-TG, and fatty acid unsaturation, as well as an integrated 51 metabolite signature. These findings extend previous MR evidence by providing complementary population based and metabolomic perspectives and suggest that systemic metabolic dysregulation may represent a plausible intermediate pathway linking MDD with CP. However, because the present study is observational, these mediation findings should be interpreted as exploratory rather than as evidence of causal mediation.

CP is traditionally associated with alcohol consumption, smoking, genetic susceptibility, and metabolic abnormalities (17, 42, 43). In the present study, smoking, physical inactivity, overweight or obesity, diabetes, and altered lipid metabolism were also more common among participants with MDD. These factors are themselves associated with pancreatic injury and CP. Thus, part of the observed association may reflect shared behavioral and metabolic susceptibility rather than a simple direct effect of MDD on the pancreas. The attenuation of the effect estimate after adjustment is consistent with this interpretation, although the remaining association indicates that conventional shared risk factors do not provide a complete explanation. The stronger association observed among participants younger than 60 years should be considered hypothesis-generating. It may reflect differences in metabolic susceptibility, exposure patterns, case ascertainment, or residual confounding and requires confirmation in independent populations.

The pancreas is particularly relevant in this shared metabolic context because it is both a potential target of metabolic injury and an important regulator of systemic energy homeostasis. Its endocrine compartment regulates glucose and nutrient metabolism through insulin, glucagon, and other hormones, whereas its exocrine compartment supports nutrient digestion and absorption. Crosstalk between these compartments is important for normal pancreatic physiology and disease (44). Obesity, insulin resistance, diabetes, and dyslipidemia may disrupt this coordination and increase susceptibility to inflammatory and lipotoxic injury (45, 46). From this perspective, part of the association between MDD and CP may reflect a shared systemic metabolic and inflammatory background rather than a unidirectional pathway from psychiatric illness to pancreatic injury.

The metabolomic findings provided more specific evidence for this shared metabolic background. Participants with MDD had higher HDL-TG and LDL-TG concentrations and a lower degree of fatty acid unsaturation. These measures were also associated with incident CP and accounted for part of the association in the exploratory mediation analyses. Experimental studies suggest that marked triglyceride-related lipid disturbances may promote pancreatic injury through lipotoxic mechanisms (47–49). Hydrolysis of triglyceride-rich lipoproteins can generate excessive unbound free fatty acids (25), which may disrupt acinar-cell membrane integrity, impair mitochondrial function, and activate pro-inflammatory signaling (50, 51). In contrast, unsaturated fatty acids, particularly polyunsaturated fatty acids, may exert anti-inflammatory and antioxidant effects that can mitigate tissue injury (27, 28). These findings provide a biologically plausible context for the HDL-TG, LDL-TG, and fatty acid unsaturation measures identified in this study. However, most supporting mechanistic evidence comes from experimental studies or acute pancreatitis models. The present data therefore cannot establish the direction of these relationships or confirm causal mediation.

Shared metabolic and behavioral characteristics may account for part of the observed association but do not fully explain the increased risk that persisted after multivariable adjustment. This suggests that, beyond the measured clinical and behavioral factors, including overweight or obesity, diabetes, smoking, and physical activity. Biological processes involving inflammation, neuroendocrine regulation, autonomic function, and central energy metabolism may also contribute to the relationship between MDD and CP. MDD is often accompanied by chronic low grade inflammation, oxidative stress, and dysregulation of the HPA axis (14, 52, 53). Abnormal glucocorticoid and catecholamine signaling may affect adipose tissue lipolysis, hepatic lipoprotein metabolism, and glucose homeostasis, while inflammatory mediators such as interleukin-6 may further promote metabolic reprogramming (54, 55). The resulting systemic inflammatory and lipotoxic environment may increase pancreatic vulnerability and provide one possible biological link between MDD and CP.

The autonomic nervous system may provide another connection between central stress responses and pancreatic function. Pancreatic islets and acinar cells receive both sympathetic and parasympathetic innervation, and autonomic signaling regulates endocrine and exocrine activity, cell survival, regeneration, and apoptosis (56). Depression related autonomic imbalance may therefore interact with circulating metabolic abnormalities to influence pancreatic secretion, metabolism, and inflammation.

Brain mitochondrial metabolism may also contribute to this brain–metabolic–pancreatic link. Neuronal energy production, redox balance, and hypothalamic control of systemic energy homeostasis all depend on normal mitochondrial function. In a recent experimental study, changes in hypothalamic mitochondrial structure and biogenesis occurred alongside changes in circulating triglycerides, body fat distribution, glycemic responses, and central energy-regulatory pathways (57). These findings suggest close links between central energy metabolism and peripheral metabolic status, although they do not establish a direct role for brain mitochondrial dysfunction in MDD associated CP.

Within this neuro–immune–metabolic framework, Toll-like receptor 4 (TLR4) related innate immune signaling may represent a context-dependent mechanism linking MDD, metabolic dysregulation, and pancreatic inflammation. Experimental studies suggest that lipopolysaccharide exposure or chronic unpredictable mild stress can enhance TLR4/MyD88/NF-κB signaling in the hippocampus and prefrontal cortex, increase IL-1β, IL-6, and TNF-α expression, and induce depression like behavior. TLR4-mediated neuroinflammation has also been associated with ultrastructural alterations within the central nervous system, providing broader support for the involvement of this pathway in neuroinflammatory processes (58). Obesity, lipid excess, and certain fatty acid related signals may further amplify TLR4 dependent inflammation (59–61). TLR4 signaling may also contribute to inflammatory amplification and tissue injury in experimental acute pancreatitis (62). However, TLR4 does not appear to be uniformly harmful across tissues or disease contexts. Genetically predicted higher TLR4 expression has been associated with a lower risk of MDD (63), whereas intestinal epithelial specific deletion of TLR4 aggravated experimental acute pancreatitis through gut dysbiosis and impaired barrier homeostasis (64). These findings highlight the context dependent effects of TLR4. Because TLR4 and its downstream signaling were not measured in this study, and the supporting evidence is mainly preclinical and based on acute pancreatitis models, this pathway should be regarded as a hypothesis requiring further investigation rather than an established causal explanation.

Beyond individual metabolites, the integrated 51-metabolite signature developed in this study further suggested that the observed association between MDD and CP may involve coordinated alterations across multiple metabolic networks rather than being driven by a single circulating biomarker (21). The apparent mediated proportion was approximately 20.8%, which decreased to 19.2% after bootstrap adjustment for in-sample optimism. Although this modest attenuation suggests some stability of the estimate, the signature was developed and evaluated within the same analytical cohort, and residual overfitting cannot be excluded. Therefore, the mediated proportion and potential predictive utility of the signature should be interpreted cautiously until externally validated in independent populations.

This study has several strengths. First, its large prospective design and long follow-up allowed assessment of the temporal association between MDD and CP while reducing the likelihood of reverse causation. Second, the integration of high-throughput NMR metabolomic data extended the analysis beyond the epidemiological association and identified specific lipid measures and coordinated metabolic patterns that may be involved. Third, the consistency of the findings across multivariable, sensitivity, and competing risk analyses supports their robustness. Several limitations should also be acknowledged. First, as an observational study, it cannot establish a causal relationship between MDD and CP. Although multiple socioeconomic, behavioral, and metabolic factors were adjusted for, residual or unmeasured confounding remains possible. In particular, detailed dietary characteristics, chronic opioid exposure, family history of pancreatitis, and several pancreatitis-related clinical conditions, including gallstone disease, autoimmune disorders, prior pancreatic surgery, and clinical hyperlipidemia, were unavailable or incompletely captured across the full analytic cohort and therefore could not be comprehensively adjusted for. Reverse causality also cannot be completely excluded because CP may develop over a prolonged subclinical period before formal diagnosis. Although the association remained materially consistent after excluding events occurring within the first 2 years and, in an additional sensitivity analysis, within the first 5 years of follow-up, these lag analyses cannot entirely rule out the possibility that undiagnosed pancreatic disease present at baseline influenced depressive status or other baseline characteristics. Second, causal interpretation of the mediation analyses requires strong assumptions, including the absence of unmeasured exposure–outcome, exposure–mediator, and mediator–outcome confounding, as well as the absence of mediator–outcome confounders affected by the exposure. These assumptions cannot be fully verified in observational data, and residual mediator–outcome confounding from incompletely measured clinical, behavioral, or genetic factors cannot be excluded. Although metabolomic measurements preceded incident CP, this temporal ordering alone is insufficient to establish causal mediation. Metabolites were measured only once at baseline, precluding assessment of longitudinal metabolic changes, and the temporal sequence between MDD onset and the identified metabolic alterations could not be precisely determined. Therefore, the estimated mediation proportions should be regarded as exploratory statistical decompositions of the observed association rather than evidence of causal biological pathways. Third, MDD was ascertained from diagnostic records and self-reported physician diagnoses, which may have introduced exposure misclassification. Individuals with subthreshold depressive symptoms or unrecorded depression may have been classified as non-MDD, potentially attenuating the observed association. Inaccuracies in diagnostic coding may also have contributed to exposure misclassification. In addition, detailed information on episode frequency, duration, and severity was not consistently available, limiting our ability to examine clinical heterogeneity in MDD. CP identified through linked health records may likewise have missed undiagnosed or milder cases. Fourth, autonomic function, brain mitochondrial metabolism, and TLR4 related signaling were not directly measured, so the corresponding mechanistic interpretations remain hypothetical. Fifth, the integrated metabolomic signature was developed and evaluated within the same analytical cohort without an independent holdout sample. Although LASSO regularization, 10-fold cross-validation, and bootstrap adjustment were used to reduce model instability and in-sample optimism, residual overfitting cannot be excluded. Accordingly, the stability, transportability, and predictive utility of the signature require validation in independent external cohorts. Finally, the UK Biobank is subject to potential selection bias because participation was voluntary and the cohort is not fully representative of the general population. Participants are predominantly of European ancestry and tend to be healthier and more socioeconomically advantaged than the source population, reflecting a healthy volunteer effect. These characteristics may influence the observed distributions of MDD, CP, and related risk factors, and may affect both the absolute incidence estimates and the magnitude of the observed association. In addition, differences in genetic background, environmental exposures, lifestyle patterns, healthcare access, and disease ascertainment may limit the applicability of our findings to Asian populations and populations living in low-income settings. Independent replication in more ethnically and socioeconomically diverse cohorts is therefore warranted before broader generalization of these findings.

In conclusion, this large prospective study found that MDD was associated with an increased risk of incident CP, with the association remaining statistically significant after multivariable adjustment. Exploratory metabolomic analyses further identified lipid-related traits, including HDL-TG, LDL-TG, and fatty acid unsaturation, together with an integrated metabolomic profile that may help characterize the metabolic processes accompanying this association. These findings extend current understanding of the relationship between mental health and CP and highlight the potential relevance of shared metabolic and behavioral risk profiles in individuals with MDD. Given the observational nature of the study, the findings should not be interpreted as evidence that MDD directly causes CP; further studies incorporating repeated metabolomic measurements, refined phenotyping, and external validation are needed to clarify the temporal and biological relationships involved.

## Data Availability

The data supporting the findings of this study are available from the UK Biobank. All researchers can access these data by following the UK Biobank application procedures.
